# The Outcomes of Gestational Diabetes Mellitus after a Telecare Approach Are Not Inferior to Traditional Outpatient Clinic Visits

**DOI:** 10.1155/2010/386941

**Published:** 2010-06-10

**Authors:** Natalia Pérez-Ferre, Mercedes Galindo, M. Dolores Fernández, Victoria Velasco, Isabelle Runkle, M. José de la Cruz, Patricia Martín Rojas-Marcos, Laura del Valle, Alfonso L. Calle-Pascual

**Affiliations:** Endocrinology and Nutrition Department, Hospital Clínico Universitario San Carlos, 28040 Madrid, Spain

## Abstract

*Objective*. To evaluate the feasibility of a telemedicine system based on Internet and a short message service in pregnancy and its influence on delivery and neonatal outcomes of women with gestational diabetes mellitus (GDM). *Methods*. 100 women diagnosed of GDM were randomized into two parallel groups, a control group based on traditional face-to-face outpatient clinic visits and an intervention group, which was provided with a Telemedicine system for the transmission of capillary glucose data and short text messages with weekly professional feedback. 97 women completed the study (48/49, resp.). *Main Outcomes Measured*. The percentage of women achieving HbA1c values <5.8%, normal vaginal delivery and having a large for-gestational-age newborn were evaluated. *Results*. Despite a significant reduction in outpatient clinic visits in the experimental group, particularly in insulin-treated women (2.4 versus 4.6 hours per insulin-treated woman resp.; *P* < .001), no significant differences were found between the experimental and traditional groups regarding HbA1c levels (all women had HbA1c <5.8% during pregnancy), normal vaginal delivery (40.8% versus 54.2%, resp.; *P* > .05) and large-for-gestational-age newborns (6.1% versus 8.3%, resp.; *P* > .05). *Conclusions*. The system significantly reduces the need for outpatient clinic visits and achieves similar pregnancy, delivery, and newborn outcomes.

## 1. Introduction

Many studies indicate that the complications of diabetes may be prevented through tight metabolic control and accurate patient monitoring [1, 2]. The active involvement of the patient in his/her management is essential to optimize results and can only be achieved if fluid and regular communication circuits are established between the patient and the healthcare providers. Traditional methods of communication via physical attendance of the patient at outpatient clinics cannot easily attain the needed level of feedback and interchange. Furthermore, the growing prevalence of diabetes makes this optimal level of assistance difficult to implement in clinical practice, given the limited health system resources available, as well as the interference that intensive follow-up can have with the working life of patients. 

Telemedicine-based systems have been increasingly used over the last decade to facilitate the monitoring of diabetes [3–5]. Different services have been designed, with varied levels of complexity, in accordance with the evolution of technology and communications engineering [6–8]. These systems are primarily designed to provide a tool to improve the quality of care through a closer communications network between the patient and the professional. Moreover, they attempt to create a more dynamic and motivating communication, involving the patients to a greater extent in their own care and making the monitoring of the disease more compatible with patients' lifestyles [6–12]. 

Women with gestational diabetes mellitus (GDM) require frequent changes in treatment and constant feedback from healthcare providers. During the short period of time up to child birth, the patient has to make important changes in nutritional habits and physical activity, undergo strict self-monitoring of capillary blood glucose, and, in some cases, must start using insulin. 

In our health area, immigration has meant a sharp increase in the number of patients with gestational diabetes, and new strategies are needed to attend these patients and avoid massification of our outpatient clinics. Our group has previously reported [13] that a telemedicine system may be used safely by women with GDM. The objective of this study is to report pregnancy, delivery, and newborn data and outcomes of women with GDM when treated using a fluid telemedicine system as compared with standard outpatient clinic care. 

## 2. Subjects, Materials, and Methods

### 2.1. Patients

We designed a prospective, randomized, clinic-based, and interventional study with two parallel groups. Eligible women diagnosed as having GDM (Carpenter-Coustan criteria) before 28 weeks of gestation and referred to the Unit of Gestational Diabetes of the Hospital Clínico Universitario San Carlos (HCSC) of Madrid, Spain, from June to December 2007 were invited to participate in the study. Sixteen women were excluded, 10 with inability to understand or to comply with the protocol and 6 women refused to participate. A total of 100 women gave their written informed consent and were allocated either to the intervention group (A, *n* = 50), provided with a telemedicine system detailed below, or allocated to the control group (B, *n* = 50) that was treated in accordance with our standard face-to-face monitoring outpatient protocol. Patients were followed until delivery. 97 women completed the study (48 from group A and 49 from group B, resp.). The study was approved by the Ethical Committee of the Hospital Clínico Universitario San Carlos and was carried out in accordance with the principles expressed in the Helsinki Declaration.

Clinical characteristics are displayed in Table 1.

### 2.2. Experimental Design

At visit 0 (between 24–28 weeks of pregnancy), patient data were collected: age, nationality, educational level, employment status, problems in access to the medical center, family history, personal history (hypertension, smoking status, obesity, thyroidal disease, and other comorbidities), obstetric history (number of pregnancies, miscarriages, and gestational diabetes in previous pregnancies), use of medications, and body weight and height. 

Patients were instructed by the nurse educator in nutritional habits and self-monitoring of capillary blood glucose and informed about the goals of glycemic control: fasting and preprandial blood glucose <95 mg/dl and 1-hour postprandial blood glucose <120 mg/dl. Body weight, blood pressure, HbA1c, and first morning urine sample albumin-to-creatinine ratio were assessed.

At visit 1, one week later (before 28 weeks gestation), capillary blood glucose values were evaluated. Six capillary blood glucose determinations a day were recommended during the first week. If more than 4 of 5 fasting and premeal glycemic values were <95 mg/dl in the first week, only 1 hour postmeal capillary blood glucose determinations were recommended daily or every other day until delivery. 

The 8 women most likely to require insulin after the evaluation of the first 7 days of blood glucose profiles (at least 50% of postmeals blood glucose values >115 mg/dl) were allocated to the telemedicine group, because this subgroup of patients was expected to need more provider contacts and therefore benefit more from the telemedicine system. 

The remaining 92 women were randomized into two groups (control and intervention), according to age and obstetric history. 

During the follow-up of both groups, 4 face-to-face visits (one a month) were scheduled until delivery (before 28 weeks of gestation (visit 1), and between 32–34 (visit 2), 36–38 (visit 3), and 39-40 weeks (visit 4)). Body weight, blood pressure, HbA1c, and first morning urine sample albumin-to-creatinine ratio were determined in each visit. Capillary glucose values recorded by the patient in her logbook were evaluated, and episodes of mild or severe hypoglycaemia and insulin requirements were registered. 

Patients in the control group were followed according to protocol for gestational diabetes at HCSC, including the same capillary blood glucose targets, and were given the opportunity to attend the outpatient clinic without prior appointment (nonscheduled visit) and bring in their logbook when their blood glucose values were above the objectives or for any queries regarding nutritional recommendations or insulin dose. The total number of patients' nonscheduled visits to the medical centre, loss of workdays, and the number of hospital admissions were regularly recorded.

### 2.3. Telemedicine System

The telemedicine system consists of a central database and peripheral units, with cellular phones and a Glucometer capable of transmitting data via infrared port. 

Each woman in the intervention group received a Glucometer (Accu-Chek Compact Plus) with a cellular phone (Nokia E50-1), the latter with a preinstalled application that allows the transmission of capillary glucose values to the central database via short message service (SMS). This application has also an interface that allows the infrared transmission of the glucose values stored in the glucometer to the cellular phone. The system enables the patient to regularly transmit blood glucose values and also to maintain contact through short text messages with health professionals as required. 

Patients were recommended to send blood glucose values recorded in the glucometer to the medical terminal once a week. 

An endocrinologist and a diabetes nurse educator evaluated patients' data accessing into the Emminens Conecta Plus Web Application (http://www.emminens.com/) from any PC with Internet connection. Entering a personal password, they had access to blood glucose values sent by the patients, accompanied by their identification by patients' initials, date and time of measurement. The application provides graphics showing glycemic trends over time, charts of everyday and weekly glucose values, and the daily glycemic values of every patient. Health professionals can send text messages from their computer, and they are received via Internet on the cellular phone of the patient. Through these messages, the professional makes recommendations for nutritional changes or adjustments in insulin doses. Patients can send text messages from their cellular phones to the medical terminal via Internet with questions as required or answer questions about their nutritional patterns or treatment. Our group previously reported that this telemedicine system may be used safely by women with GDM [13].

### 2.4. Statistical Analysis

Sample size was estimated to test the hypothesis that the telemedicine-based intervention would not be inferior to standard therapy. A primary endpoint difference was detecting more than a 20% difference in HbA1c of patients achieving HbA1c values <5.8%. With 40 patients in each group, the study had 80% power to detect a 20% difference between groups at 5% significance.

The statistical study was performed by using SPSS 15.0 program for Windows. Descriptive data are expressed as median and Q1-Q3 or mean ± SDM. Nonparametric Mann-Whitney and Kruskal-Wallis tests were carried out to detect significant differences between groups.

## 3. Results

There was a significant 62% reduction in outpatient clinic visits in women from the telemedicine group. This reduction was even greater in the case of the insulin-treated women of the group, with a 82% reduction of outpatient women (*P* < .03) as compared with those patients who exclusively assisted in the outpatient clinic setting. Women from the telemedicine group achieved similar HbA1c values (all <5.8%), systolic and diastolic blood pressure values, albumin-to-creatinine ratio, and weight gain (5.820 + 3.950 versus 6.446 + 4.988 Kg; *P* > .05) as the control patients. The average total number of outpatient clinic visits was 9.11 for insulin-treated diabetic women in the control group (4.6 hours per woman) as compared to 4.25 for those in the intervention group (2.37 hours per insulin-treated woman). In addition to the outpatient clinic visits, insulin-treated women from the intervention group had an additional 10.8 contacts on average (1.44 hours per insulin-treated woman). In other words, the women in the intervention group had more contacts with health personnel (15.05 versus 9.11) taking up less time (3.8 versus 4.6 hours; *P* < .001) than the control group. Data are displayed in Table 2. Pregnancy-induced hypertension was observed in 2 women from the telemedicine group. Normal vaginal birth rate (20 (40.8%) versus 26 (54.2%), resp.; *P* < .068) and the frequency of caesarean section (17 (34.7%) versus 12 (25%), resp.; *P* > .05) were similar in both groups 3 (6.1%) and 4 (8.3%). A similar percentage of neonates were large for gestational age in both groups (6,1 and 8,3 % resp.). Data are displayed in Table 3. We did not detect differences in clinical and laboratory data during the follow-up nor were differences in delivery and neonatal outcomes observed. 

## 4. Discussion

The present study shows that a telemedicine system can be useful as an alternative to traditional outpatient clinic visits. Although women in the telemedicine group attended the outpatient clinic less frequently than those in the control group, we found no deleterious effects on metabolic control, pregnancy, delivery, or on the newborn despite the presence of a higher proportion of insulin-using patients in the telemedicine group.

A reduction in the number of clinic visits saves time of both the patient and the health professional, and the telemedicine connection increases patient accessibility to the professional team, permitting contact at the women's convenience. Needless to say, telemedicine communications facilitate patients' lives, reducing transportation and outpatient waiting times and minimizing interfere with patients' regular work schedules. The telemedicine system was particularly useful in the subgroup of insulin-treated patients, who require more contacts to adjust the insulin dose. The extent of the use of the telemedicine system has been variable and is highly dependent on the woman's cultural level [13]. However, in our study, cultural level was similar in both groups. At visit 1, all the patients assigned to the intervention group received information about the system and were asked to send capillary blood glucose values at least once a week. Access to a computer was not required in order to do so. Some women in the intervention group, particularly those with a lower cultural level, preferred directly attending the outpatient clinic or the use of a landline phone for questions they considered important.

The professional team also made use of land phones at certain times, particularly upon initiation of insulinization or when correct patient compliance was in doubt. But most treatment decisions involving control of glycemia after initiation of insulin therapy did not require outpatient clinic attendance. Insulin dose adjustment can be based on the data of home blood glucose monitoring each 5 profiles (3 out of 5, 1 hour post meal values >120 mg/dl), as used in our study). Therefore doses could be adjusted every 5 days.

The median outpatient visit duration was 30 minutes whereas the telematic visit lasted less than 8 minutes, including assessment of capillary blood glucose profiles and SMS response. In our study, the telemedicine system not only made attention more convenient for the patient, it was also was less expensive for the health system in terms of use of health professionals' time. Similarly others studies [14–17] on women from the telemedicine group indicate a higher level of adherence to treatment and increased satisfaction. 

Although the telemedicine group had a higher rate of C-sections, this difference was no significant. Furthermore, there was no between-group difference in metabolic control, pregnancy duration, fetal outcome, or neonatal morbidity. In addition, gestational week at delivery, preterm birth, fetal macrosomia, and neonatal morbidity were similar to mothers with normal glucose tolerance during pregnancy in our hospital. These data suggest that the algorithms used in the study for management of gestational diabetes mellitus are useful in order to reach similar outcomes of pregnancy of women without gestational diabetes mellitus.

Despite the potential benefits, the use of telemedicine is still very limited and is not integrated into healthcare systems. There are a few small-scale projects designed to study the usefulness of telemedicine in patients with GDM [18–20]. The Commission of the European Communities recently has written a recommendation [21] to encourage Member States in an effort to integrate these new services into healthcare systems, focusing on improving the confidence and acceptance of telemedicine services through consistent studies of effectiveness and cost-effectiveness, as well as bringing legal clarity, solving technical issues, and facilitating market development, promoting the interoperability of the systems, and improving their quality and safety.

We conclude that a telemedicine system can be a useful tool in the treatment of gestational diabetes patients, as a complement to conventional outpatient clinic visits, especially in cases requiring tighter glycemic control or with difficulties in access to the medical centre [22]. 

## Figures and Tables

**Table 1:** Characteristics of the survey population by groups.

	Control	Telemedicine	*P*
N	48	49	

Age (years)	34.19 ± 5.18	33.33 ± 5.58	.357

Race/Ethnicity			.608
Caucasian	27 (56.3%)	25 (51%)	
Hispanic	18 (37.5%)	15 (30.6%)	
Asian	2 (4.2%)	3 (6.1%)	
North African	1 (2.1%)	2 (4.1%)	
Others	0 (0%)	4 (8.2%)	

Education			.188
Below high school	9 (18.8%)	8 (16.3%)	
Some high school	12 (25%)	5 (10.2%)	
High school graduate	9 (18.8%)	14 (28.6%)	
College or above	6 (12.5%)	9 (18.4%)	
Unknown	12 (25%)	13 (26.5%)	

EMPLOYMENT	28 (58.3%)	27 (55.1)	.371

Access problems to office	31 (64.6%)	28 (57.1%)	.490

Family history of Diabetes	23 (47.9%)	23 (46.9%)	.494

Number of pregnancies	2.48 ± 1.51	2.06 ± 1.36	.162
Primiparous	12 (25%)	18 (38.3%)	
Second pregnancy	19 (39.6%)	19 (40.4%)	
>2 pregnancies	17 (35.4%)	10 (21.3%)	

MISCARRIAGE	19 (39.6%)	13 (26.5%)	.215
Prior GDM	9 (18.8%)	4 (8.2%)	.104
Hypertension	5 (10.4%)	4 (8.2%)	.590
Thyroid disease	7 (14.6%)	8 (16.3%)	.361
Current smoker	2 (4.2%)	3 (6.1%)	.081
HbA1c at entry (%)	5.10 ± 0.41	5.03 ± 0.38	.164
Prepregnancy body weight (kg)	74.06 ± 15.37	70.46 ± 12.98	.470
Prepregnancy BMI (kg/m^2^)	29.01 ± 5.74	27.96 ± 5.24	.588

Data are Mean ± SDM or *n* (%).

GDM denotes Gestational Diabetes Mellitus; BMI: Body Mass Index.

**Table 2:** Maternal Metabolic parameters during gestation.

		Control group	Telemedicine group
sBP (mm Hg)	Visit 1	122.0 ± 16.8	122.3 ± 12.5
	Visit 2	122.3 ± 14.5	120.8 ± 11.1
	Visit 3	121.9 ± 13,2	125.1 ± 9.8
	Visit 4	120.8 ± 14.8	122.9 ± 10.8

dBP (mm Hg)	Visit 1	71.5 ± 8.6	72.6 ± 9.5
	Visit 2	71.4 ± 8.6	72.8 ± 5.6
	Visit 3	72.3 ± 9.1	74.6 ± 8.9
	Visit 4	72.1 ± 8.0	76.8 ± 10.6

HbA1c (%)	Visit 1	5.2 ± 0.4	5.0 ± 0.4
	Visit 2	5.2 ± 0.4	5.0 ± 0.3
	Visit 3	5.3 ± 0.4	5.2 ± 0.4
	Visit 4	5.4 ± 0.4	5.3 ± 0.4

Albumin-to-creatinine Ratio (mg/g)	Visit 1	6.8 ±4.9	10.1 ±14.4
	Visit 2	6.4 ± 3.8	7.8 ± 5.6
	Visit 3	8.2 ± 7.9	10.0 ± 8.6
	Visit 4	5.1 ± 2.9	7.7 ± 5.3

Body Weight (Kg)	Visit 1	76.9 ± 14.3	75.9 ± 13.2
	Visit 2	77.9 ± 14.8	76.8 ± 11.8
	Visit 3	78.6 ± 15.8	77.8 ± 12.9
	Visit 4	82.3 ± 16.3	80.7 ± 14.7

Weight Gain (Kg)	Visit 1–4	6.446 ± 4.988	5.822 ± 3.950

Insulin-treated Women *n* (%)		9 (18.8%)	17 (34.7%)
Total contact per Insulin-treated women (total hours)		9.11 (4.6)	15.05 (3.8)***

Data expressed as mean ± SDM. sBP, systolic blood pressure. dBP, diastolic blood pressure. ACR, first morning urine sample albumin-to-creatinine ratio.

***P < .001.

**Table 3:** Gestation, Delivery and New Born data.

	Control group	Telemedicine group	*P*
N	48	49	
Gestational Weeks at Delivery	39.42 ± 1.42	39.12 ± 1.66	n.s.
Pregnancy induced hypertension	0 (0%)	2 (4.1%)	.501
Delivery Outcomes			
Normal vaginal birth	26 (54.2%)	20 (40.8%)	.068
Dystocia	17 (35.4%)	27 (55.1%)	
(i) Caesarean Section	12 (25%)	17 (34.7%)	.427
(ii) Instrumental vaginal birth	5 (10.4%)	10 (20.4%)	
New born gender (M/F)	22 (47.9%)/18 (37.5%)	20 (40.8%)/26 (53.1%)	.240
Birth weight (g)	3370.6 ± 479.1	3308.2 ± 488.8	.385
(i) Male	3407.1 ± 492.2	3214.5 ± 435.7	
(ii) Female	3346.9 ± 481.3	3380.2 ± 522.9	
New born Outcomes			.500
Large-for-gestational age	4 (8.3%)	3 (6.1%)	
Hypoglycemia	0 (0%)	1 (2%)	
Hypokaliemia	0	0	
Hypocalcemia	0	0	
Poliglobulia	0	0	
Small-for-gestational age	0	0	
Preterm Birth (GA <37 weeks)	1 (2.1%)	1 (2.0%)	
Loss of fetal wellbeing	5 (10.4%)	3 (6.1%)	
Umbilical cord pathology	2 (4.2%)	1 (2.0%)	
Shoulders dystocia	1 (2.1%)	0 (0%)	
Abruptio placentae	1 (2.1%)	0 (0%)	

Data are Mean ± SDM or *n* (%).
